# Comment on: “Complications Associated with Initial Clinical Presentation of Cystic Echinococcosis: A 20-year Cohort Analysis”

**DOI:** 10.4269/ajtmh.19-0741a

**Published:** 2020-01

**Authors:** Tommaso Manciulli, Maria Teresa Giordani, Raffaella Lissandrin, Enrico Brunetti, Francesca Tamarozzi

**Affiliations:** PhD School of Experimental Medicine; University of Pavia; Pavia, Italy; Department of Clinical-Surgical; Diagnostic and Pediatric Sciences; University of Pavia; Pavia, Italy; E-mail: tommaso.manciulli01@ateneopv.it; Infectious and Tropical Diseases Department; San Bortolo Hospital; Vicenza, Italy; E-mail: giordanimt@libero.it; Department of Clinical-Surgical; Diagnostic and Pediatric Sciences; University of Pavia; Pavia, Italy; E-mail: raffaella.lissandrin@unipv.it; Department of Clinical-Surgical; Diagnostic and Pediatric Sciences; University of Pavia; Pavia, Italy; Unit of Infectious and Tropical Diseases; IRCCS Fondazione Policlinico San Matteo; Pavia, Italy; E-mail: enrico.brunetti@unipv.it; WHO Collaborating Centre for Epidemiology; Detection and Control of Cystic and Alveolar Echinococcosis; Department of Infectious Diseases; Foodborne and Neglected Parasitic Diseases Unit, Istituto Superiore di Sanità; Rome, Italy; E-mail: f_tamarozzi@yahoo.it

Dear Sir,

We read with interest the article by Collado-Aliaga et al.^1^ published in the recent 101(3) issue of *Am J Trop Med Hyg*. The authors tried to identify risk factors for complications of cystic echinococcosis (CE) using retrospective hospital data retrieved through ICD-9 codes from a single tertiary hospital in Spain.^2^

We think that the article has several limitations.

The first issue is the rather fuzzy definition of “complications.” In the article, the presence of “clinical, radiological or microbiological symptoms or signs attributable to CE” makes a cyst complicated. However, such definition is unclear and it is exceedingly difficult to attribute unspecific symptoms from retrospective records. Pain in patients with CE may have a different cause than CE, and other “clinical manifestations” associated with complications listed in Figure 2 are vague (e.g., “general syndrome”) or have no proper meaning (“urinary clinic” and “tumoration”). Had the authors divided their cohort into asymptomatic uncomplicated, symptomatic uncomplicated, and symptomatic complicated patients, a more plausible picture of complicated CE in their cohort would have been provided.

The second issue is the methodology used. It is unclear whether the study is cross-sectional (if so, the time points at which extracted patients data refer to are missing) or longitudinal (in which case, the length of follow-up, and difference in the median/mean follow-up length and rate of loss-to-follow-up between groups are missing). It is also unclear which groups were compared in the different parts of the study, as well as when head-to-head comparisons and when multivariable analysis were applied. Furthermore, the authors indicate that about 15% of patients were excluded from the analysis because of “missing information,” but did not specify how they accounted for this high percentage of missing data. Patients with complications related to treatment were also excluded from the analysis. This choice is debatable, as these may well have been asymptomatic for a variable time (before treatment) and, therefore, could have been included in the asymptomatic group for the pretreatment follow-up period. The authors also fail to indicate the number of patients in this category.

The authors found no difference in the distribution of complications across cyst stage or size; therefore, they conclude that neither variable should be considered to decide on surgery or other treatments. However, it is unclear on what groups comparison analysis the authors based this conclusion, and the biases in the patient cohort (selection bias deriving from using hospitalized patients records, bias deriving from follow-up differences between groups and from exclusion of patients) hamper any generalization of this type. Calculating the rate of complications starting from an uncomplicated state, stratified by cyst stage, may be a more correct approach. Also, “watch and wait” is a misnomer here. With this term, the WHO-IWGE refers exclusively to patients with uncomplicated and asymptomatic inactive CE4 and CE5 cysts,^3–6^ and not to “…the elderly with other comorbidities.”^1^ This alone may account for the surprisingly high rate of “complications” recorded in patients with inactive cysts.

In conclusion, we think this article has serious limitations that may invalidate some conclusions.
